# Supplementary material for: Drug Delivery Systems: Study of Inclusion Complex Formation between Methylxanthines and Cyclodextrins and Their Thermodynamic and Transport Properties
Source: Biomolecules. 2019 May 20;9(5):196. doi: 10.3390/biom9050196 (PMC6571592; doi:10.3390/biom9050196)
Supplement: Supplementary file 1 [file biomolecules-09-00196-s001.pdf]

Table S1: Densities and apparent molar volumes for caffeine aqueous solutions at various molalities at 298.15 K and 310.15 K.

m / (10^{-3} mol kg^{-1})	ρ / (g cm^{-3}) (298.15 K)	$10^6\Delta$ (a)	ϕ_V / (cm^3 mol^{-1})	ρ / (g cm^{-3}) (310.15 K)	$10^6\Delta$ (a)	ϕ_V / (cm^3 mol^{-1})
0.50572	0.997073	1.5	145.0_3	0.993355	1.0	151.4_0
1.01145	0.997099	1.2	144.0_4	0.993379	5.6	149.3_9
4.04580	0.997257	2.2	142.7_7	0.993528	1.0	146.6_2
8.09159	0.997468	3.8	142.4_9	0.993727	2.5	146.0_9
10.3145	0.997584	6.7	142.4_1	0.993836	7.5	145.9_6
12.6431	0.997706	3.2	142.3_6	0.993951	1.5	145.8_6
25.2862	0.998363	2.5	142.2_8	0.994574	4.1	145.5_7
50.5725	0.999665	2.1	142.3_4	0.995817	1.8	145.3_5

[(a)] Δ stands for standard deviation of all the measurements; u(m) = 5x10^{-8} mol kg^{-1}; u(ρ) = 5x10^{-6} g cm^{-3}

Table S2: Experimental viscosity data for caffeine aqueous solutions at various molalities at 298.15 K and 310.15 K.

m /(10^{-3} mol kg^{-1})	η /(mPa s) (298.15 K)	$10^3\Delta$ (a)	η /(mPa s) (310.15 K)	$10^3\Delta$ (a)
0.50572	0.8903	0.7	0.6928	0.9
1.01145	0.8906	0.7	0.6933	0.8
4.04580	0.8912	0.3	0.6952	0.5
8.09159	0.8921	0.5	0.6977	0.2
10.3145	0.8928	0.2	0.6998	0.3
12.6431	0.8933	0.7	0.7014	0.3
25.2862	0.8967	0.5	0.7104	0.2
50.5725	0.9040	0.3	0.7283	0.3

[(a)] Δ stands for standard deviation of all the measurements; u(m)= 5x10^{-8} mol kg^{-1}; u(η)= 5x10^{-4} mPa s.

Table S3: Coefficient values obtained from the analysis of the viscosity-concentration dependence for caffeine aqueous solutions.

Temperature / K	A / (dm$^{3/2}$ mol$^{-1/2}$)	B / (dm^3 mol^{-1})	r^2
298.15	- 0.010 (±0.002)	0.345 (±0.009)	0.999
310.15	- 0.004 (±0.006)	1.04_8 (±0.03)	0.999

Table S4: Binary mutual diffusion coefficients of aqueous solutions of caffeine measured with the Taylor technique at 298.15 K and 310.15 K.

c /(mol dm^{-3})	Δc /(mol dm^{-3})	D [a] /(10^{-9} m^2 s^{-1}) [a,12] (298.15 K)	$D^a \pm S_D$ [b] /(10^{-9} m^2 s^{-1}) [b,c] (310.15 K)
0.001	0.001	0.760	1.053 ±0.012
0.002	0.002	0.751	1.031 ±0.009
0.004	0.004	0.742	0.995 ±0.008
0.005	0.004	0.738	0.980 ±0.009
0.008	0.004	0.719	0.952 ±0.005
0.010	0.004	0.703	0.944 ±0.003

(a) Each tabulated value of D corresponds to an average of at least 4 replicates; (b) S_D represents the standard deviation of the mean.

Table S5: Densities and apparent molar volumes for theophilline aqueous solutions at various molalities at 298.15 K and 310.15 K.

m / (10^{-3} mol kg^{-1})	ρ / (g cm^{-3}) (298.15 K)	$10^6\Delta$ (a)	ϕ_V / (cm^3 mol^{-1})	ρ / (g cm^{-3}) (310.15 K)	$10^6\Delta$ (a)	ϕ_V / (cm^3 mol^{-1})
0.506551	0.997067	1.0	143.62	0.993350	1.0	147.62
1.01310	0.997087	2.3	142.29	0.993369	1.6	144.72
2.02621	0.997124	1.9	142.95	0.993408	3.5	143.74
4.05241	0.997207	1.5	141.30	0.993479	4.1	144.68
8.30482	0.997386	4.0	139.75	0.993643	3.9	143.42
10.1310	0.997466	3.1	139.10	0.993734	4.0	141.23
15.1965	0.997672	4.2	139.32	0.993917	3.8	142.32
20.2621	0.997887	2.3	138.93	0.994128	1.6	141.46
40.5241	0.998708	1.1	139.26	0.994899	4.5	141.97

(a) Δ stands for standard deviation of all the measurements; u(m) = 5x10^{-8} mol kg^{-1}; u(ρ) = 5x10^{-6} g cm^{-3}.

Table S6: Experimental viscosity data for theophylline aqueous solutions at various molalities at 298.15 K and 310.15 K.

m /(10^{-3} mol kg^{-1})	η /(mPa s) (298.15 K)	$10^3\Delta$ (a)	η /(mPa s) (310.15 K)	$10^3\Delta$ (a)
0.506551	0.8904	1.0	0.6916	0.7
1.01310	0.8905	2.3	0.6917	0.4
2.02621	0.8907	1.9	0.6924	0.6
4.05241	0.8914	1.5	0.6946	0.6
8.30482	0.8919	4.0	0.6961	0.4
10.1310	0.8922	3.1	0.6974	0.9
15.1965	0.8934	4.2	0.7002	0.7
20.2621	0.8955	2.3	0.7042	0.3
40.5241	0.8996	1.1	0.7174	0.5

[(a)] Δ stands for standard deviation of all the measurements; u(m)= 5x10^{-8} mol kg^{-1}; u(η)= 5x10^{-4} mPa s.

Table S7: Coefficient values obtained from the analysis of the viscosity-concentration dependence for caffeine aqueous solutions.

Temperatura / K	A / (kg mol$^{-1/2}$)	B / (kg^2 mol$^{-3/2}$)	r^2
298.15	- 0.012 (±0.001)	0.309 (±0.008)	0.999
310.15	- 0.021 (±0.007)	1.031 (±0.032)	0.999

Table S8: Binary mutual diffusion coefficients of aqueous solutions of theophylline measured with the Taylor technique at 298.15 K and 310.15 K.

c /(mol dm^{-3})	Δc /(mol dm^{-3})	$D^{a} \pm S_D{}^{b}$ /(10^{-9} m^2 s^{-1}) (298.15 K)	$D^{a} \pm S_D{}^{b}$ /(10^{-9} m^2 s^{-1}) (310.15 K)
0.001	0.001	0.801 ± 0.012	1.124 ± 0.009
0.002	0.002	0.785 ± 0.007	1.098 ± 0.009
0.004	0.004	0.769 ± 0.005	1.053 ± 0.009
0.005	0.004	0.762 ± 0.005[c]	1.035 ± 0.009[c]
0.008	0.004	0.742 ± 0.006	0.994 ± 0.005
0.010	0.004	0.734 ± 0.004[c]	0.979 ± 0.006[c]

[(a)] Each tabulated value of D corresponds to an average of at least 4 replicates; [(b)] S_D represents the standard deviation of the mean; [(c)] reference [68].

Table S9: Densities and apparent molar volumes for β-cyclodextrin aqueous solutions at various molalities at 298.15 K and 310.15 K.

m / (10^{-3} mol kg^{-1})	ρ / (g cm^{-3}) (298.15 K)	10$^6\Delta$ (a)	ϕ_V / (cm^3 mol^{-1})	ρ / (g cm^{-3}) (310.15 K)	10$^6\Delta$ (a)	ϕ_V / (cm^3 mol^{-1})
0.43659	0.997236	8.4	705.0_4	0.993513	3.2	724.6_3
0.89532	0.997433	4.8	705.9_5	0.993705	3.1	721.8_0
1.74313	0.997796	5.2	706.3_8	0.994059	2.6	720.2_6
3.47949	0.998537	2.2	706.8_1	0.994783	3.8	719.2_7
6.95898	1.00001_7	2.8	707.0_2	0.996229	4.6	718.6_9
8.73180	1.00076_9	2.9	707.0_3	0.996966	5.9	718.3_6
10.5840	1.00155_3	3.3	706.9_8	0.997732	4.9	718.1_6
13.2300	1.00267_1	3.3	706.8_2	0.998823	4.2	718.0_7

[(a)] Δ stands for standard deviation of all the measurements; u(m) = 5x10^{-8} mol kg^{-1}; u(ρ) = 5x10^{-6} g cm^{-3}.

Table S10: Experimental viscosity data for β-cyclodextrin aqueous solutions at various molalities at 298.15 K and 310.15 K.

m /(10^{-3} mol kg^{-1})	η /(mPa s) (298.15 K)	$10^3\Delta$ (a)	η /(mPa s) (310.15 K)	$10^3\Delta$ (a)
0.43659	0.8909	0.7	0.6934	0.2
0.89532	0.8917	0.7	0.6944	0.8
1.74313	0.8931	0.3	0.6962	0.5
3.47949	0.8961	1.0	0.6998	0.2
6.95898	0.9019	0.8	0.7071	0.6
8.73180	0.9049	0.3	0.7109	0.3
10.5840	0.9080	0.5	0.7148	0.2
13.2300	0.9125	0.3	0.7203	0.3

(a) Δ stands for standard deviation of all the measurements, u(m) = 5x10^{-8} mol kg^{-1}; u(η) = 5x10^{-4} mPa s.

Table S11: Coefficient values obtained from the analysis of the viscosity-concentration dependence for β-cyclodextrin aqueous solutions.

Temperature / K	B / (dm^3 mol^{-1})	D / (dm^6 mol^{-2})	r^2
298.15	1.89 ±0.01	-0.044 ±0.80	0.999
310.15	3.05 ±0.02	-0.36 ±1.2	0.999

Table S12: Binary mutual diffusion coefficients of aqueous solutions of β-CD measured with the Taylor technique at 298.15 K and 310.15 K.

c /(mol dm^{-3})	Δc /(mol dm^{-3})	$D^a \pm S_D{}^b$ /(10^{-9} m^2 s^{-1}) (298.15 K)^c	$D^a \pm S_D{}^b$ /(10^{-9} m^2 s^{-1}) (310.15 K)
0.002	0.002	0.324 ±0.003	0.443 ±0.005
0.004	0.004	0.323 ±0.001	0.441 ±0.004
0.005	0.004	0.322 ±0.003	0.441 ±0.003
0.006	0.004	0.321 ±0.003	0.440 ±0.003
0.008	0.004	0.318 ±0.002	0.439 ±0.004
0.010	0.004	0.316 ±0.002	0.436 ±0.004

(a) Each tabulated value of D corresponds to an average of at least 4 replicates; (b) S_D represents the standard deviation of the mean; (c) reference [75].

Table S13: Densities and apparent molar volumes for HP-β-CD aqueous solutions at various molalities at 298.15 K and 310.15 K.

m / (10^{-3} mol kg^{-1})	ρ / (g cm^{-3}) (298.15 K)	$10^6\Delta$ (a)	ϕ_V / (cm^3 mol^{-1})	ρ / (g cm^{-3}) (310.15 K)	$10^6\Delta$ (a)	ϕ_V / (cm^3 mol^{-1})
0.414638	0.997266	2.1	854.21	0.993543	2.7	874.49
0.826814	0.997478	0.5	860.56	0.993750	4.1	877.69
1.64707	0.997894	0.5	866.46	0.994150	5.6	885.54
3.31054	0.998772	0.7	858.84	0.994981	3.5	883.38
6.62107	1.00040$_0$	2.8	871.86	0.996610	5.2	884.71
8.27634	1.00127$_4$	1.0	866.72	0.997459	5.8	880.38
12.4145	1.00334$_3$	1.0	868.55	0.999400	3.2	888.53
16.5363	1.00549$_0$	0.4	863.24	1.00159$_2$	4.2	875.82

[(a)] Δ stands for standard deviation of all the measurements; u(m) = 5x10^{-8} mol kg^{-1}; u(ρ) = 5x10^{-6} g cm^{-3}.

Table S14: Experimental viscosity data for HP-β-CD aqueous solutions at various molalities at 298.15 K and 310.15 K.

10^3 m /(mol kg^{-1})	η /(mPa s)	10^3 Δ (a)	η /(mPa s)	10^3 Δ (a)
	T = 298.15 K		T = 310.15 K	
0.414638	0.8912	1.0	0.6930	1.2
0.826814	0.8920	0.5	0.6945	0.8
1.64707	0.8936	0.5	0.6974	0.7
3.31054	0.8969	0.7	0.7033	0.8
6.62107	0.9034	2.8	0.7152	0.6
8.27634	0.9066	1.0	0.7211	0.9
12.4145	0.9148	1.0	0.7359	0.6
16.5363	0.9228	0.4	0.7507	0.4

[(a)] Δ stands for standard deviation of all the measurements, u(m) = 5x10^{-8} mol kg^{-1}; u(η) = 5x10^{-4} mPa s.

Table S15: Coefficient values obtained from the analysis of the viscosity-concentration dependence for HP-β-CD aqueous solutions.

Temperature / K	B / (dm^3 mol^{-1})	D / (dm^6 mol^{-2})	r^2
298.15	2.20 ($\pm 2 \cdot 10^{-13}$)	- 3.1 $\cdot 10^{-12}$ ($\pm 1 \cdot 10^{-11}$)	0.999
310.15	5.19 ($\pm 8 \cdot 10^{-13}$)	- 4.0 $\cdot 10^{-12}$ ($\pm 5 \cdot 10^{-11}$)	0.999

Table S16: Binary mutual diffusion coefficients of aqueous solutions of HP-β-CD measured with the Taylor technique at 298.15 K and 310.15 K.

c /(mol dm^{-3})	Δc /(mol dm^{-3})	$D^{a} \pm S_D{}^{b}$ /(10^{-9} m^2 s^{-1}) (298.15 K)	$D^{a} \pm S_D{}^{b}$ /(10^{-9} m^2 s^{-1}) (310.15 K)
0.002	0.002	0.319 ±0.003	0.408 ±0.006
0.004	0.004	0.316 ±0.001	0.407 ±0.003
0.005	0.004	0.314 ±0.002	0.407 ±0.003
0.008	0.004	0.310 ±0.002	0.406 ±0.002
0.010	0.008	0.307 ±0.002	0.406 ±0.004

[(a)] Each tabulated value of D corresponds to an average of at least 4 replicates; [(b)] S_D represents the standard deviation of the mean.

Table S17: Densities, viscosities and apparent molar volumes for caffeine + β-CD + water ternary systems at various concentrations at 298.15 K and 310.15 K.

		T = 298.15 K			T = 310.15 K		
$10^3\ m_{\beta\text{-}CD}$ /(mol kg^{-1})	$10^3\ m_{cafeína}$ /(mol kg^{-1})	ρ /(g cm^{-3})	η /(mPa s)	ϕ_V /(cm^3 mol^{-1})	ρ /(g cm^{-3})	η /(mPa s)	ϕ_V /(cm^3 mol^{-1})
0.465996	5.05840	0.997279	0.8912	143.34	0.993553	0.6937	145.96
0.465996	1.01168	0.997305	0.8913	143.28	0.993578	0.6938	145.91
0.465996	4.04672	0.997462	0.8927	142.68	0.993727	0.6945	145.74
0.465996	8.06119	0.997675	0.8942	142.24	0.993927	0.6954	145.54
0.465996	10.1168	0.997783	0.8949	141.98	0.994027	0.6958	145.47
0.931992	5.05840	0.997478	0.8924	141.22	0.993748	0.6946	143.67
0.931992	1.01168	0.997505	0.8930	141.12	0.993774	0.6947	143.61
0.931992	4.04672	0.997668	0.8950	140.78	0.993929	0.6956	143.50
0.931992	8.09343	0.997886	0.8973	140.56	0.994138	0.6968	143.31
0.931992	10.1168	0.997997	0.8983	140.37	0.994243	0.6975	143.25
3.70056	5.05840	0.998658	0.8971	127.68	0.994899	0.7004	140.35
3.70056	1.01168	0.998689	0.8977	128.16	0.994927	0.7006	140.30
3.70056	4.04672	0.998873	0.9009	130.20	0.995092	0.7016	140.19
3.70056	8.09343	0.999117	0.9039	131.74	0.995311	0.7029	140.31
3.70056	10.1168	0.999240	0.9049	132.04	0.995422	0.7037	140.17
7.26405	5.05840	1.000176	0.9031	131.58	0.996381	0.7080	137.46
7.26405	1.01168	1.000209	0.9038	133.15	0.996409	0.7081	137.50
7.26405	4.04672	1.000401	0.9076	131.73	0.996583	0.7090	137.43
7.26405	8.09343	1.000647	0.9111	133.93	0.996811	0.7105	137.68
7.26405	10.1168	1.000770	0.9122	132.38	0.996928	0.7112	137.44
9.18286	5.05840	1.000958	0.9065	122.80	0.997180	0.7120	133.39
9.18286	1.01168	1.000994	0.9073	123.67	0.997210	0.7121	133.44
9.18286	4.04672	1.001029	0.9120	126.07	0.997396	0.7132	133.24
9.18286	8.09343	1.001233	0.9167	128.66	0.997642	0.7146	133.42
9.18286	10.1168	1.001487	0.9183	129.37	0.997765	0.7154	133.39

u(m) = 5x10^{-8} mol kg^{-1}; u(ρ) = 5x10^{-6} g cm^{-3}; u(η) = 5x10^{-4} mPa s; u(T) < 0.02 K.

Table S18: Densities, viscosities and apparent molar volumes for caffeine + HP-β-CD + water ternary systems at various concentrations at 298.15 K and 310.15 K.

		T = 298.15 K			T = 310.15 K		
10^3 $m_{\beta\text{-CD}}$ /(mol kg^{-1})	10^3 $m_{cafe\acute{n}a}$ /(mol kg^{-1})	ρ /(g cm^{-3})	η /(mPa s)	ϕ_V /(cm^3 mol^{-1})	ρ /(g cm^{-3})	η /(mPa s)	ϕ_V /(cm^3 mol^{-1})
0.417997	0.505840	0.997297		137.16	0.993559		160.48
0.417997	1.01168	0.997319		144.27	0.993586		150.52
0.417997	4.04672	0.997472		143.97	0.993737		146.39
0.417997	8.09343	0.997675		144.13	0.993938		145.72
0.417997	10.1168	0.997772		144.60	0.994030		146.43
0.835994	0.505840	0.997509		141.60	0.993768		155.93
0.835994	1.01168	0.997534	0.8929	143.90	0.993791	0.6952	152.98
0.835994	4.04672	0.997681	0.8954	145.35	0.993941	0.6966	147.06
0.835994	8.09343	0.997880	0.8973	145.23	0.994134	0.6984	147.09
0.835994	1.01168	0.997989	0.8982	144.25	0.994249	0.6990	145.20
3.34398	0.505840	0.998782		143.79	0.995012		152.67
3.34398	1.01168	0.998803	0.8988	147.41	0.995038	0.7049	148.64
3.34398	4.04672	0.998951	0.9026	145.98	0.995183	0.7082	147.33
3.34398	8.09343	0.999144	0.9060	146.36	0.995378	0.7119	146.85
3.34398	1.01168	0.999255	0.9068	144.91	0.995492	0.7130	145.13
6.68795	0.505840	1.00047_8		145.71	0.996671		149.53
6.68795	1.01168	1.00050_2	0.9060	146.79	0.996697	0.7174	145.91
6.68795	4.04672	1.00064_6	0.9117	146.65	0.996844	0.7223	146.11
6.68795	8.09343	1.00083_8	0.9162	146.66	0.997042	0.7267	145.81
6.68795	1.01168	1.00095_0	0.9172	145.04	0.997147	0.7279	145.12
8.35994	0.505840	1.00132_5		150.31	0.997502		144.59
8.35994	1.01168	1.00135_1	0.9108	146.49	0.997528	0.7235	143.80
8.35994	4.04672	1.00149_3	0.9189	146.97	0.997677	0.7293	144.83
8.35994	8.09343	1.00168_8	0.9254	146.42	0.997875	0.7342	145.17
8.35994	1.01168	1.00179_7	0.9265	145.17	0.997979	0.7359	144.76

u(m) = 5x10^{-8} mol kg^{-1}; u(ρ) = 5x10^{-6} g cm^{-3}; u(η) = 5x10^{-4} mPa s; u(T) < 0.02 K.

Table S19: Partial molar volumes, ϕ_V^0, and transfer volumes, $\Delta\phi_V^0$, for aqueous solutions of caffeine in the presence of cyclodextrins at 298.15 K and 310.15 K.

$10^3\ m_{\beta\text{-}CD}$ /(mol kg^{-1})	ϕ_V^0 /(cm^3 mol^{-1})	$\Delta\phi_V^0$ /(cm^3 mol^{-1})	$10^3\ m_{HP\text{-}\beta\text{-}CD}$ /(mol kg^{-1})	ϕ_V^0 /(cm^3 mol^{-1})	$\Delta\phi_V^0$ /(cm^3 mol^{-1})
			T = 298.15 K		
0.000000	142.5 (±0.2)				
0.465996	143.79 (±0.06)	1.3	0.417997	139.1 (±2.8)	-4.40
0.931992	141.46 (±0.03)	-1.0	0.835994	142.2 (±1.3)	-1.30
3.70056	133.69 (±0.04)	-8.8	3.34398	145.5 (±1.6)	2.04
7.26405	126.39 (±0.15)	-16.1	6.68795	146.6 (±0.8)	3.07
9.18286	120.89 (±0.16)	-21.6	8.35994	149.7 (±1.5)	6.21
			T = 310.15 K		
0.000000	146.5 (±0.2)				
0.465996	146.11 (±0.02)	-0.4	0.417997	158.9 (±4.2)	10.44
0.931992	143.80 (±0.03)	-2.7	0.835994	157.4 (±1.7)	8.92
3.70056	140.35 (±0.07)	-6.1	3.34398	152.6 (±1.5)	4.16
7.26405	137.45 (±0.11)	-9.0	6.68795	148.8 (±1.4)	0.30
9.18286	133.39 (±0.09)	-13.1	8.35994	144.0 (±0.4)	-4.46

Note: The values in parentheses correspond to the standard deviations of the measurements.

Table S20: Thermodynamic properties of the complexation process of cyclodextrins with caffeine at 298.15 K and 310.15 K.

	K /(kg mol^{-1})	$\phi_{V,c\,Caf}$ /(cm^3 mol^{-1})	$\Delta\phi_{V,c\,Caf}$ /(cm^3 mol^{-1})	$\phi_{V,c\,CD}$ /(cm^3 mol^{-1})	$\Delta\phi_{V,c\,CD}$ /(cm^3 mol^{-1})
			T = 298.15 K		
β-CD	47.89	123.0 (±3.1)	-19.50	668.2 (±7.3)	-38.07
HP-*β-CD*	187.9	145.3 (±0.1)	2.77	875.1 (±1.8)	6.71
			T = 310.15 K		
β-CD	25.15	130.4 (±2.8)	-16.40	655.5 (±3.8)	-62.57
HP-*β-CD*	--	144.7 (±0.4)	-1.98	882.3 (±0.1)	0.84

Note: The values in parentheses correspond to the standard deviations of the measurements.

Table S21: Coefficients *A* and *B* of the Jones-Dole equation and transfer Δ*B* coefficients of the aqueous solutions of caffeine in the presence of cyclodextrins at 298.15 K and 310.15 K.

$10^3\ m_{\beta\text{-}CD}$ /(mol dm^{-3})	*A* / (dm$^{3/2}$ mol$^{-1/2}$)	*B* / (dm^3 mol^{-1})	*ΔB* / (dm^3 mol^{-1})	$10^3\ m_{\text{HP-}\beta\text{-}CD}$ (mol dm^{-3})	*A* / (dm$^{3/2}$ mol$^{-1/2}$)	*B* / (dm^3 mol^{-1})	*ΔB* / (dm^3 mol^{-1})
T = 298.15 K							
0.000000	-0.010	0.345					
0.465996	0.003	0.413	0.068	0.417997			
0.931992	0.029	0.445	0.100	0.835994	0.027	0.428	0.118
3.70056	0.039	0.589	0.244	3.34398	0.062	0.499	0.188
7.26405	0.043	0.700	0.355	6.68795	0.093	0.629	0.319
9.18286	0.044	1.00_8	0.663	8.35994	0.151	0.741	0.431
T = 310.15 K							
0.000000	-0.004	1.04_8					
0.465996	0.006	0.273	-0.775	0.417997			
0.931992	-0.005	0.474	-0.574	0.835994	0.018	0.470	-0.568
3.70056	-0.003	0.510	-0.538	3.34398	0.044	0.937	-0.101
7.26405	-0.005	0.524	-0.524	6.68795	0.081	0.976	-0.062
9.18286	-0.003	0.528	-0.520	8.35994	0.085	1.19_1	0.153

Table S22: Ternary mutual diffusion coefficients D_{11}, D_{12}, D_{21} and D_{22} for aqueous β-CD (1) + caffeine (2) solutions and respective standard deviations, S_D, at 298.15 K and 310.15 K.

c_1 [a]	c_2 [a]	$D_{11} \pm S_D$ [b] /10^{-9} ($m^2 s^{-1}$)	$D_{12} \pm S_D$ /10^{-9} ($m^2 s^{-1}$)	$D_{21} \pm S_D$ /10^{-9} ($m^2 s^{-1}$)	$D_{22} \pm S_D$ /10^{-9} ($m^2 s^{-1}$)	D_{12}/D_{22} [b]	D_{21}/D_{11} [c]
T = 298.15 K							
0.002	0.002	0.323 ±0.017	-0.008 ±0.003	0.016 ±0.008	0.735 ±0.022	-0.011	0.048
0.004	0.004	0.319 ±0.016	-0.009 ±0.003	0.015 ±0.007	0.728 ±0.019	-0.012	0.047
0.005	0.005	0.318 ±0.017	-0.011 ±0.003	0.014 ±0.005	0.725 ±0.017	-0.015	0.043
0.008	0.008	0.315 ±0.007	-0.010 ±0.003	0.014 ±0.003	0.715 ±0.015	-0.014	0.044
0.000	0.010	0.322 ±0.004	0.002 ±0.001	0.064 ±0.010	0.721 ±0.011	0.003	0.199
0.010	0.000	0.321 ±0.004	-0.040 ±0.001	-0.004 ±0.004	0.746 ±0.025	-0.054	-0.013
0.010	0.002	0.315 ±0.007	-0.033 ±0.003	-0.051 ±0.012	0.703 ±0.022	-0.047	-0.162
0.010	0.005	0.313 ±0.006	-0.039 ±0.005	-0.001 ±0.011	0.699 ±0.017	-0.056	-0.003
0.010	0.010	0.313 ±0.007	-0.014 ±0.003	0.013 ±0.002	0.695 ±0.013	-0.020	0.041
T = 310.15 K							
0.002	0.002	0.443 ±0.007	-0.017 ±0.012	0.031 ±0.008	1.009 ±0.016	-0.017	0.070
0.005	0.005	0.441 ±0.007	0.006 ±0.035	0.051 ±0.033	0.958 ±0.011	0.007	0.116
0.010	0.002	0.429 ±0.008	-0.005 ±0.021	0.012 ±0.017	0.999 ±0.022	-0.005	0.028
0.010	0.005	0.428 ±0.013	-0.008 ±0.024	0.037 ±0.020	0.937 ±0.015	-0.009	0.085
0.010	0.010	0.424 ±0.008	0.020 ±0.011	0.054 ±0.031	0.868 ±0.012	0.023	0.127

[(a)] c_1 and c_2 in units (mol dm^{-3}); [(b)] D_{12}/D_{22} represents the number of moles of β-CD carried by 1 mole of caffeine; [(c)] D_{21}/D_{11} represents the number of moles of caffeine carried by 1 mole of β-CD.

Table S23: Ternary mutual diffusion coefficients D_{11}, D_{12}, D_{21} and D_{22} for aqueous HP-β-CD (1) + caffeine (2) solutions and respective standard deviations, S_D, at 298.15 K and 310.15 K.

c_1 [a]	c_2 [a]	$D_{11} \pm S_D$ [b] /10^{-9} ($m^2 s^{-1}$)	$D_{12} \pm S_D$ /10^{-9} ($m^2 s^{-1}$)	$D_{21} \pm S_D$ /10^{-9} ($m^2 s^{-1}$)	$D_{22} \pm S_D$ /10^{-9} ($m^2 s^{-1}$)	D_{12}/D_{22} [b]	D_{21}/D_{11} [c]
T = 298.15 K							
0.000_5	0.000_5	0.305 ±0.010	-0.003 ±0.015	0.040 ±0.025	0.744 ±0.043	-0.004	0.131
0.001	0.001	0.300 ±0.015	0.003 ±0.014	0.050 ±0.026	0.730 ±0.033	0.004	0.167
0.002_5	0.005	0.316 ±0.004	-0.004 ±0.004	0.067 ±0.013	0.676 ±0.016	-0.006	0.212
0.005	0.002_5	0.292 ±0.003	-0.003 ±0.013	0.051 ±0.015	0.690 ±0.039	-0.017	0.184
0.005	0.005	0.293 ±0.006	-0.011 ±0.006	0.054 ±0.024	0.665 ±0.013	-0.004	0.175
0.000	0.010	0.322 ±0.004	-0.002 ±0.003	0.031 ±0.003	0.738 ±0.003	-0.003	0.097
0.010	0.000	0.321 ±0.003	0.020 ±0.003	0.004 ±0.003	0.600 ±0.003	0.033	0.014
0.010	0.002	0.289 ±0.006	-0.042 ±0.023	0.024 ±0.015	0.665 ±0.012	-0.063	0.083
0.010	0.005	0.288 ±0.008	-0.040 ±0.016	0.043 ±0.011	0.646 ±0.013	-0.069	0.148
0.010	0.010	0.283 ±0.009	-0.021 ±0.016	0.103 ±0.022	0.640 ±0.036	-0.033	0.160
T = 310.15 K							
0.002	0.002	0.407 ±0.015	-0.038 ±0.047	0.070 ±0.061	0.997 ±0.029	-0.038	0.172
0.005	0.005	0.406 ±0.006	0.057 ±0.035	0.080 ±0.033	0.883 ±0.013	0.065	0.197
0.010	0.002	0.403 ±0.009	0.077 ±0.040	0.031 ±0.033	0.875 ±0.011	0.088	0.077
0.010	0.005	0.403 ±0.004	0.061 ±0.023	0.033 ±0.013	0.852 ±0.018	0.072	0.081
0.010	0.010	0.401 ±0.013	0.024 ±0.014	0.113 ±0.031	0.811 ±0.012	0.030	0.282

[(a)] c_1 and c_2 in units (mol dm^{-3}); [(b)] D_{12}/D_{22} represents the number of moles of HP-β-CD carried by 1 mole of caffeine; [(c)] D_{21}/D_{11} represents the number of moles of caffeine carried by 1 mole of HP-β-CD.

Table S24: Values of K and D_{33}^* estimated from the ternary diffusion coefficients of caffeine aqueous solutions in the presence of β-CD at 298.15 K and 310.15 K.

c_2 /(mol dm^{-3})	D_{11}^* /10^{-9} (m^2 s^{-1})	D_{22}^* /10^{-9} (m^2 s^{-1})	D_{33}^* /10^{-9} (m^2 s^{-1})	K /(dm^3 mol^{-1})
		T = 298.15 K		
0.002		0.737		
0.005	0.317	0.724	0.305 (±0.016)	43 (± 5.6)
0.010		0.69		
		T = 310.15 K		
0.002		0.999		
0.005	0.436	0.949	0.430 (± 0.018)	23 (± 2.1)
0.010		0.915		

Note: The data presented corresponds to a constant concentration of 10 mM β-CD.

Table S25: Values of K and D_{33}^* estimated from the ternary diffusion coefficients of aqueous solutions of caffeine in the presence of HP-β-CD at 298.15 K and 310.15 K.

c_2 /(mol dm^{-3})	D_{11}^* /10^{-9} (m^2 s^{-1})	D_{22}^* /10^{-9} (m^2 s^{-1})	D_{33}^* /10^{-9} (m^2 s^{-1})	K /(dm^3 mol^{-1})
		T = 298.15 K		
0.002		0.735		
0.005	0.307	0.723	0.300 (± 0.013)	85 (± 8.6)
0.010		0.688		
		T = 310.15 K		
0.002		0.979		
0.005	0.406	0.930	0.395 (± 0.018)	20 (±1.0)
0.010		0.896		

Note: The data presented are those corresponding to a constant concentration of 10 mM HP-β-CD.

Table S26: Densities, viscosities and apparent molar volumes for theophylline + β-CD + water ternary systems at various concentrations at 298.15 K and 310.15 K.

		T = 298.15 K			T = 310.15 K		
$10^3\ m_{\beta\text{-}CD}$ /(mol kg^{-1})	$10^3\ m_{teofilina}$ /(mol kg^{-1})	ρ /(g cm^{-3})	η / (mPa s) (298.15 K)	ϕ_V /(cm^3 mol^{-1})	ρ /(g cm^{-3})	η / (mPa s) (310.15 K)	ϕ_V /(cm^3 mol^{-1})
0.438541	0.508594	0.997261		136.08	0.993544		137.49
0.441887	1.01682	0.997288		132.05	0.993573		130.75
0.439680	4.04695	0.997460		125.58	0.993740		126.84
0.439680	8.09389	0.997678		125.95	0.993950		127.81
0.439680	10.1174	0.997794		125.37	0.994063		127.14
0.884839	0.513092	0.997451		130.97	0.993730		131.84
0.882710	1.01559	0.997481	0.8925	125.44	0.993759	0.6934	126.69
0.878307	4.04210	0.997664	0.8940	121.23	0.993932	0.6940	124.36
0.878307	8.08421	0.997890	0.8963	122.81	0.994143	0.6952	126.35
0.878307	10.1053	0.998024	0.8977	121.04	0.994268	0.6959	124.75
3.51816	0.509203	0.998583		124.52	0.994831		127.73
3.54503	1.01804	0.998618	0.8971	118.50	0.994862	0.6991	123.63
3.52730	4.05180	0.998816	0.8998	115.70	0.995027	0.6999	125.31
3.52730	8.10361	0.999057	0.9026	118.21	0.995260	0.7013	124.13
3.52730	10.1295	0.999199	0.9039	116.59	0.995383	0.7023	123.19
7.03631	0.509203	1.00008_9		120.53	0.996295		125.86
7.05461	1.01295	1.00012_2	0.9034	117.93	0.996326	0.7065	122.61
7.05461	4.05180	1.00032_2	0.9067	115.23	0.996497	0.7072	123.67
7.05461	8.10361	1.00057_2	0.9014	116.77	0.996717	0.7087	124.86
7.05461	10.1295	1.00069_7	0.9119	117.05	0.996842	0.7097	123.59
8.83948	0.511755	1.00084_3		117.49	0.997031		118.08
8.81826	1.01295	1.00087_7	0.9071	114.51	0.997057	0.7102	122.92
8.81826	4.05180	1.00108_0	0.9116	113.54	0.997237	0.7107	121.71
8.81826	8.10361	1.00132_9	0.9162	116.13	0.997464	0.7124	122.89
8.81826	10.1295	1.00145_4	0.9178	116.56	0.997577	0.7132	123.26

Table S27: Densities, viscosities and apparent molar volumes for theophylline + HP-β-CD + water ternary systems at various concentrations at 298.15 K and 310.15 K.

		T = 298.15 K			T = 310.15 K		
$10^3\ m_{\beta\text{-}CD}$ /(mol kg^{-1})	$10^3\ m_{teofilina}$ /(mol kg^{-1})	ρ /(g cm^{-3})	η / (mPa s) (298.15 K)	ϕ_V /(cm^3 mol^{-1})	ρ /(g cm^{-3})	η / (mPa s) (310.15 K)	ϕ_V /(cm^3 mol^{-1})
0.419669	0.505612	0.997293		136.69	0.993566		134.04
0.419669	1.00320	0.997323		128.62	0.993599		123.73
0.419669	4.01280	0.997495		124.47	0.993771		123.61
0.419669	8.02560	0.997716		124.88	0.993989		124.89
0.419669	10.0320	0.997834		124.20	0.994118		123.05
0.839338	0.505612	0.997510		130.29	0.993777		126.69
0.839338	1.00320	0.997537	0.8930	127.30	0.993809	0.6954	121.87
0.839338	4.01280	0.997714	0.8958	123.08	0.993982	0.6976	122.65
0.839338	8.02560	0.997948	0.8982	122.52	0.994217	0.6998	122.33
0.839338	10.0320	0.998064	0.8991	122.47	0.994330	0.7004	122.72
3.35735	0.505612	0.998789		127.99	0.995026		124.62
3.35735	1.00320	0.998822	0.8989	121.19	0.995060	0.7050	118.55
3.35735	4.01280	0.999001	0.9036	120.79	0.995238	0.7089	120.46
3.35735	8.02560	0.999226	0.9078	122.44	0.995492	0.7124	118.84
3.35735	10.0320	0.999341	0.9092	122.58	0.995594	0.7137	120.93
6.71470	0.505612	1.00049_5		126.03	0.996691		122.34
6.71470	1.00320	1.00052_8	0.9061	119.60	0.996725	0.7175	117.03
6.71470	4.01280	1.00071_2	0.9122	119.12	0.996915	0.7223	117.13
6.71470	8.02560	1.00094_1	0.9174	121.14	0.997166	0.7271	117.42
6.71470	10.0320	1.00106_3	0.9187	120.66	0.997266	0.7283	120.01
8.39338	0.505612	1.00134_9		123.22	0.997523		121.70
8.39338	1.00320	1.00138_2	0.9104	117.80	0.997558	0.7240	115.85
8.39338	4.01280	1.00156_1	0.9181	120.04	0.997738	0.7302	119.35
8.39338	8.02560	1.00179_8	0.9249	120.51	0.997994	0.7357	117.81
8.39338	10.0320	1.00190_5	0.9261	121.68	0.998109	0.7374	118.83

Table S28: Partial molar volumes, ϕ_V^0, and partial molar transfer volumes, $\Delta\phi_V^0$, for aqueous solutions of theophylline in the presence of cyclodextrins at 298.15 K and 310.15 K.

$10^3\ m_{\beta\text{-}CD}$ /(mol kg^{-1})	ϕ_V^0 /(cm^3 mol^{-1})	$\Delta\phi_V^0$ /(cm^3 mol^{-1})	$10^3\ m_{HP\text{-}\beta\text{-}CD}$ /(mol kg^{-1})	ϕ_V^0 /(cm^3 mol^{-1})	$\Delta\phi_V^0$ /(cm^3 mol^{-1})
T = 298.15 K					
0.000000	143.2 (±0.7)				
0.439680	136.8 (±2.4)	-6.33	0.4196690	135.6 (±3.5)	-7.57
0.878307	130.4 (±2.7)	-12.75	0.8393380	130.9 (±1.5)	-12.21
3.527304	122.9 (±2.9)	-20.25	3.357352	125.3 (±3.0)	-17.86
7.054608	119.87 (±1.7)	-23.40	6.714704	123.5 (±2.9)	-19.67
8.818260	115.5 (±1.8)	-27.62	8.393380	120.4 (±2.3)	-22.77
T = 310.15 K					
0.000000	145.9 (±0.9)				
0.439680	136.5 (±2.5)	-9.48	0.4196690	131.0 (±4.2)	-14.90
0.878307	130.6 (±2.5)	-15.34	0.8393380	125.1 (±1.9)	-20.84
3.527304	126.9 (±1.6)	-17.04	3.357352	122.4 (±2.6)	-23.52
7.054608	124.4 (±1.4)	-21.51	6.714704	119.8 (±2.6)	-26.10
8.818260	119.2 (±1.8)	-26.76	8.393380	119.4 (±2.4)	-26.55

Note: The values in parentheses correspond to the standard deviations of the measurements.

Table S29: Thermodynamic properties of the complexation process of cyclodextrins with theophylline at 298.15 K and 310.15 K.

	K /(kg mol^{-1})	$\phi_{V,cTeof}$ /(cm^3 mol^{-1})	$\Delta\phi_{V,cTeof}$ /(cm^3 mol^{-1})	$\phi_{V,cCD}$ /(cm^3 mol^{-1})	$\Delta\phi_{V,cCD}$ /(cm^3 mol^{-1})
T = 298.15 K					
β-CD	30.53	115.6 (±0.5)	-24.87	596.4 (±43.1)	-109.82
HP-*β-CD*	78.71	121.3 (±0.5)	-19.17	813.5 (±18.3)	-51.22
T = 310.15 K					
β-CD	16.56	122.9 (±0.3)	-20.31	568.7 (±78.2)	-149.38
HP-*β-CD*	48.47	119.7 (±0.6)	-23.56	787.9 (±66.3)	-93.64

Table S30: Coefficients *A* and *B* of the Jones-Dole equation and transfer Δ*B* coefficients for the aqueous solutions of theophylline in the presence of cyclodextrin at 298.15 K and 310.15 K.

10^3 $m_{\beta\text{-}CD}$ /(mol dm^{-3})	*A* / (dm$^{3/2}$ mol$^{-1/2}$)	*B* / (dm^3 mol^{-1})	*ΔB* / (dm^3 mol^{-1})	10^3 $m_{\text{HP-}\beta\text{-}CD}$ (mol dm^{-3})	*A* / (dm$^{3/2}$ mol$^{-1/2}$)	*B* / (dm^3 mol^{-1})	*ΔB* / (dm^3 mol^{-1})
T = 298.15 K							
0.000000	-0.012	0.3094	0	0.000000			0
0.878307	-0.005	0.689	0.380	0.839338	0.003	0.535	0.226
3.527304	0.014	0.707	0.398	3.357352	0.059	0.800	0.491
7.054608	0.018	0.894	0.585	6.714704	0.087	0.862	0.553
8.818260	0.049	0.923	0.613	8.393380	0.127	0.935	0.626
T = 310.15 K							
0.000000	-0.021	1.028	0	0.000000			0
0.878307	-0.019	0.538	-0.490	0.839338	0.043	0.545	-0.483
3.527304	-0.017	0.626	-0.402	3.357352	0.054	0.942	-0.086
7.054608	-0.020	0.647	-0.382	6.714704	0.073	1.110	0.082
8.818260	-0.023	0.652	-0.376	8.393380	0.098	1.284	0.256

Table S31: Ternary mutual diffusion coefficients D_{11}, D_{12}, D_{21} and D_{22} for aqueous β-CD (1) + theophylline (2) solutions and respective standard deviations, S_D, at 298.15 K and 310.15 K.

c_1 [a]	c_2 [a]	$D_{11} \pm S_D$ [b] /10^{-9} ($m^2 s^{-1}$)	$D_{12} \pm S_D$ /10^{-9} ($m^2 s^{-1}$)	$D_{21} \pm S_D$ /10^{-9} ($m^2 s^{-1}$)	$D_{22} \pm S_D$ /10^{-9} ($m^2 s^{-1}$)	D_{12}/D_{22} [b]	D_{21}/D_{11} [c]
T = 298.15 K							
0.002	0.002	0.321 ±0.009	-0.003 ±0.017	0.004 ±0.005	0.721 ±0.032	-0.005	0.012
0.002	0.005	0.322 ±0.007	-0.006 ±0.017	0.003 ±0.017	0.709 ±0.030	-0.008	0.009
0.002	0.010	0.319 ±0.009	-0.005 ±0.019	0.006 ±0.008	0.680 ±0.027	-0.007	0.019
0.005	0.005	0.316 ±0.005	0.005 ±0.023	-0.024 ±0.040	0.683 ±0.034	0.008	-0.075
0.000	0.010	0.323 ±0.009	-0.001 ±0.021	0.022 ±0.006	0.722 ±0.011	-0.002	0.068
0.010	0.000	0.315 ±0.014	-0.085 ±0.024	-0.001 ±0.036	0.805 ±0.011	-0.105	-0.005
0.010	0.002	0.310 ±0.005	-0.039 ±0.016	-0.029 ±0.040	0.709 ±0.021	-0.055	-0.093
0.010	0.005	0.305 ±0.048	-0.033 ±0.110	-0.053 ±0.052	0.640 ±0.077	-0.052	-0.174
0.010	0.010	0.309 ±0.006	-0.016 ±0.030	-0.063 ±0.022	0.628 ±0.017	-0.026	-0.202
T = 310.15 K							
0.002	0.002	0.436 ±0.008	0.005 ±0.009	0.033 ±0.006	0.991 ±0.007	0.005	0.077
0.005	0.005	0.430 ±0.012	0.020 ±0.011	-0.065 ±0.022	0.898 ±0.001	0.023	-0.151
0.010	0.002	0.429 ±0.009	0.040 ±0.024	-0.060 ±0.019	0.901 ±0.005	0.044	-0.139
0.010	0.005	0.424 ±0.009	0.032 ±0.017	-0.038 ±0.017	0.863 ±0.008	0.037	-0.090
0.010	0.010	0.417 ±0.006	0.022 ±0.010	-0.048 ±0.016	0.795 ±0.002	0.028	-0.116

(a) c_1 and c_2 in units (mol dm^{-3}); (b) D_{12}/D_{22} represents the number of moles of β-CD carried by 1 mole of theophylline; (c) D_{21}/D_{11} represents the number of moles of theophylline carried by 1 mole of β-CD.

Table S32: Ternary mutual diffusion coefficients D_{11}, D_{12}, D_{21} and D_{22} for aqueous HP-β-CD (1) + theophylline (2) solutions and respective standard deviations, S_D, at 298.15 K and 310.15 K.

c_1 [a]	c_2 [a]	$D_{11} \pm S_D$ [b] /10^{-9} (m^2 s^{-1})	$D_{12} \pm S_D$ /10^{-9} (m^2 s^{-1})	$D_{21} \pm S_D$ /10^{-9} (m^2 s^{-1})	$D_{22} \pm S_D$ /10^{-9} (m^2 s^{-1})	D_{12}/D_{22} [b]	D_{21}/D_{11} [c]
T = 298.15 K							
0.002	0.002	0.306 ±0.002	0.002 ±0.001	0.068 ±0.020	0.756 ±0.017	0.003	0.222
0.002	0.005	0.305 ±0.003	0.002 ±0.003	0.070 ±0.022	0.736 ±0.009	0.003	0.230
0.002	0.010	0.304 ±0.008	-0.002 ±0.001	0.042 ±0.016	0.713 ±0.005	-0.002	0.138
0.005	0.002	0.303 ±0.007	0.008 ±0.008	0.054 ±0.019	0.719 ±0.008	0.011	0.178
0.005	0.005	0.301 ±0.006	0.024 ±0.001	0.063 ±0.024	0.682 ±0.026	0.035	0.211
0.005	0.010	0.300 ±0.007	-0.010 ±0.002	0.020 ±0.028	0.641 ±0.020	-0.016	0.067
0.000	0.010	0.303 ±0.005	0.009 ±0.011	0.032 ±0.007	0.644 ±0.009	0.002	0.107
0.010	0.000	0.300 ±0.003	0.052 ±0.014	0.006 ±0.011	0.673 ±0.013	0.077	0.020
0.010	0.002	0.300 ±0.007	0.014 ±0.005	0.042 ±0.002	0.597 ±0.026	0.023	0.140
0.010	0.005	0.299 ±0.008	0.023 ±0.004	0.056 ±0.053	0.570 ±0.034	0.040	0.187
0.010	0.010	0.298 ±0.005	-0.029 ±0.019	-0.009 ±0.090	0.548 ±0.038	-0.053	-0.031
T = 310.15 K							
0.002	0.002	0.411 ±0.004	-0.041 ±0.007	0.102 ±0.072	1.054 ±0.026	-0.039	0.248
0.005	0.005	0.408 ±0.005	-0.006 ±0.001	0.039 ±0.023	0.923 ±0.010	-0.006	0.095
0.010	0.002	0.406 ±0.006	-0.043 ±0.015	0.077 ±0.039	0.904 ±0.013	-0.047	0.189
0.010	0.005	0.406 ±0.004	-0.018 ±0.012	0.053 ±0.018	0.866 ±0.004	-0.021	0.130
0.010	0.010	0.406 ±0.008	-0.013 ±0.008	0.235 ±0.046	0.856 ±0.020	-0.015	0.578

[(a)] c_1 and c_2 in units (mol dm^{-3}); [(b)] D_{12}/D_{22} represents the number of moles of HP-β-CD carried by 1 mole of theophylline; [(c)] D_{21}/D_{11} represents the number of moles of theophylline carried by 1 mole of HP-β-CD.

Table S33: Values of K and D_{33}^* estimated from the ternary diffusion coefficients of the aqueous solutions of theophylline in the presence of β-cyclodextrin at 298.15 K and 310.15 K.

c_2 /(mol dm^{-3})	D_{11}^* /10^{-9} (m^2 s^{-1})	D_{22}^* /10^{-9} (m^2 s^{-1})	D_{33}^* /10^{-9} (m^2 s^{-1})	K /(dm^3 mol^{-1})
		T = 298.15 K		
0.002		0.770		
0.005	0.317	0.748	0.300 (±0.012)	28.5 (±1.4)
0.010		0.720		
		T = 310.15 K		
0.002		1.064		
0.005	0.436	1.003	0.395 (±0.027)	22 (±2.3)
0.010		0.948		

Note: The data presented are those corresponding to a constant concentration of 10 mM β-CD.

Table S34: Values of K and D_{33}^* estimated from the ternary diffusion coefficients of the aqueous solutions of theophylline in the presence of HP-β-cyclodextrin at 298.15 K and 310.15 K.

c_2 /(mol dm^{-3})	D_{11}^* /10^{-9} (m^2 s^{-1})	D_{22}^* /10^{-9} (m^2 s^{-1})	D_{33}^* /10^{-9} (m^2 s^{-1})	K /(dm^3 mol^{-1})
		T = 298.15 K		
0.002		0.769		
0.005	0.307	0.746	0.265 (±0.013)	85 (±8.6)
0.010		0.719		
		T = 310.15 K		
0.002		1.042		
0.005	0.406	0.982	0.385 (±0.012)	39 (±1.4)
0.010		0.929		

Note: The data presented are those corresponding to a constant concentration of 10 mM HP-β-CD.
